# *Nitrosopumilus* as main source of isoprenoid glycerol dialkyl glycerol tetraether lipids in the central Baltic Sea

**DOI:** 10.3389/fmicb.2023.1216130

**Published:** 2023-09-28

**Authors:** Anna Katharina Wittenborn, Thorsten Bauersachs, Christiane Hassenrück, Katja Käding, Janine Wäge-Recchioni, Klaus Jürgens, Helge Wolfgang Arz, Jérôme Kaiser

**Affiliations:** ^1^Marine Geology, Leibniz Institute for Baltic Sea Research – Warnemünde (IOW), Warnemünde, Germany; ^2^Institute of Earth Sciences, Heidelberg University, Heidelberg, Germany; ^3^Biological Oceanography, Leibniz Institute for Baltic Sea Research – Warnemünde (IOW), Warnemünde, Germany

**Keywords:** Thaumarchaeota, Nitrososphaeria, intact and core GDGTs, TEX86, 16S rRNA, CARD-FISH, suspended particulate matter

## Abstract

Nitrososphaeria in the phylum Crenarchaeota, is a widespread archaeal class in the oceanic realm, playing an important role in the marine carbon and nitrogen cycle. Nitrososphaeria-derived membrane lipids, i.e., isoprenoid glycerol dialkyl glycerol tetraethers (GDGTs), are commonly employed to reconstruct past water temperatures using the TetraEther indeX of 86 carbon atoms (TEX_86_). This index is of particular importance for the brackish Baltic Sea as to date it appears to be the only applicable organic temperature proxy. In this study, we investigated the distribution of intact and core GDGTs and their potential source organisms in the water column of three deep basins located in the central Baltic Sea to evaluate the application of TEX_86_. A lipidomic approach on suspended particulate matter was combined with the molecular techniques 16S rRNA gene amplicon sequencing and CARD-FISH. The archaeal community was dominated by *Nitrosopumilus* (~83–100% of the total archaeal sequences). As other detected taxa known to produce GDGTs each represented less than 2% of the total archaeal sequences, *Nitrosopumilus* is likely the most dominant GDGT producer in the central Baltic Sea. However, the occurrence of phosphohexose (PH), instead of hexose-phosphohexose (HPH) headgroups, suggested that *Nitrosopumilus* in the Baltic Sea may differ physiologically from representatives of marine settings and other marginal seas, such as the Black Sea. In the Baltic Sea, *Nitrosopumilus* is most abundant in the suboxic zone, where intact cells peak according to both CARD-FISH data and intact polar lipid concentrations. The presented data therefore suggest that TEX_86_ reflects subsurface rather than surface temperature in the central Baltic Sea.

## 1. Introduction

Archaea, one of the three domains of life, are present in all environments worldwide. They play an important role in the marine carbon and nitrogen cycle, especially Crenarchaeota which represent up to 20% of all picoplankton cells in the world’s oceans (Karner et al., 2001; Schattenhofer et al., 2009). All characterized Thaumarchaeota [now the class Nitrososphaeria (Rinke et al., 2021) in the phylum Crenarchaeota according to SILVA release 138.1 (Quast et al., 2013)], are chemolithoautotrophic ammonia-oxidizers, i.e., they gain energy by the oxidation of ammonia to nitrite and form simple organic molecules by the fixation of CO_2_ (Stahl and de la Torre, 2012; Offre et al., 2013; Könneke et al., 2014).

Some representatives of Crenarchaeota and Euryarchaeota biosynthesize isoprenoid glycerol dialkyl glycerol tetraethers (GDGTs) as membrane lipids (Schouten et al., 2012, 2013; Bauersachs et al., 2015). The backbone of the GDGT structure is the core lipid, usually consisting of 0 to 4 cyclopentane rings (abbreviated as GDGT-0, GDGT-1, GDGT-2, GDGT-3 and GDGT-4). Nitrososphaeria further produce a GDGT with an additional cyclohexane ring, known as crenarchaeol (cren; Sinninghe Damsté et al., 2002). In the archaeal cell membranes, these core GDGTs are connected via a phosphate ester bond or a glycosidic ether bond to one or two polar headgroups to form a diverse suite of intact polar lipids (IPLs; Sturt et al., 2004). Such IPL-GDGTs represent biomarkers for living cells (Schouten et al., 2010, 2012; Lengger et al., 2012) because they are prone to rapid degradation after cell lysis and do not preserve well in the geological record (Harvey et al., 1986; Schouten et al., 2010).

The Nitrososphaeria membrane lipid composition is strongly affected by temperature. Increasing temperature enhances the amount of cyclopentane rings in the GDGT structure (Wuchter et al., 2004; Schouten et al., 2007a,b). This relationship is used to calculate the TetraEther indeX of 86 carbon atoms (TEX_86_; Schouten et al., 2002) and its derivatives to reconstruct past ocean water temperatures (Huguet et al., 2006; Jonas et al., 2017). The TEX_86_, however, may be influenced by factors other than temperature, such as, e.g., community composition (Kim et al., 2016; Elling et al., 2017; Besseling et al., 2019; Sollai et al., 2019; Rattanasriampaipong et al., 2022) and oxygen concentration (Qin et al., 2015). In addition, archaea other than Nitrososphaeria such as Thermococcales (class Thermococci) are able to produce GDGTs, and it was suggested that the ability to synthesize tetraethers could have been transferred to taxonomically related species (Tourte et al., 2020). Furthermore, some species of Nitrososphaeria might be deep dwelling and their GDGT composition might not indicate a sea surface temperature (SST) signal but a mixed or even a subsurface temperature signal (Taylor et al., 2013; Kim et al., 2015; Besseling et al., 2019). Thus, it is of crucial importance to identify the potential sources and the depth of GDGT origin in a given environment in order to apply the TEX_86_ temperature proxy with confidence. Recent research showed that TEX_86_-derived temperatures extracted from sediments of the central Baltic Sea were best correlated with subsurface water temperatures (Wittenborn et al., 2022) and not summer SSTs as previously suggested (Kabel et al., 2012). In order to evaluate the application of the TEX_86_ and other GDGT-based indices for the Baltic Sea, measurements of IPL-GDGTs and core GDGTs were conducted in conjunction with molecular data analysis to explore the archaeal community composition and origin of GDGTs in the Baltic Sea water column.

## 2. Materials and methods

### 2.1. Working area

**Figure 1:**
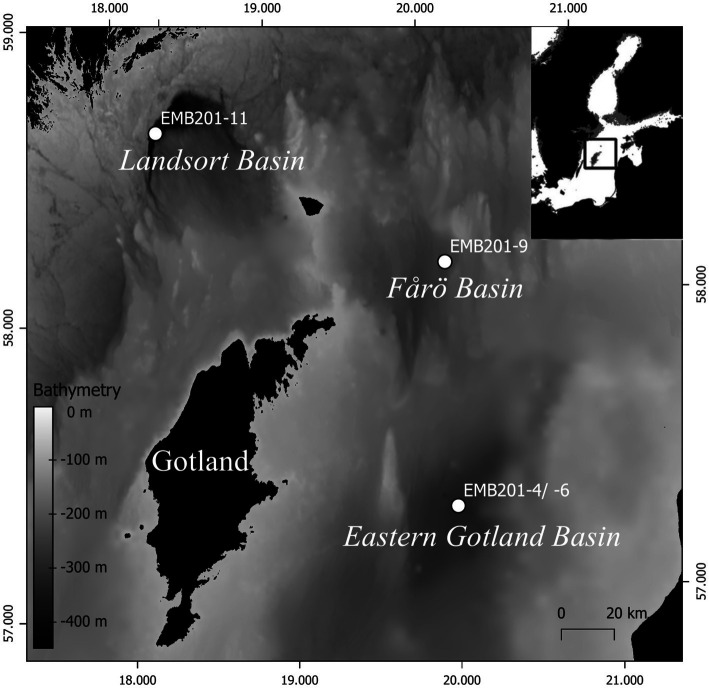
Suspended particulate matter sampling sites in the central Baltic Sea. Inset: location of the study area.

The Baltic Sea is the world’s largest brackish inland sea (Snoeijs-Leijonmalm and Andrén, 2017). Its average water depth is 55 m, but it contains multiple basins that are significantly deeper. These include, among others, the Fårö Basin (205 m), the East Gotland Basin (EGB; 250 m) and the Landsort Deep (460 m; Figure 1) in the central Baltic Sea. The deep basins are characterized by a strong stratification formed by continuous freshwater discharge from the Baltic Sea drainage area and occasional inflows of oxygen-rich, saline water from the North Sea (Meier et al., 2006; Mohrholz et al., 2015). This situation results in oxygen-rich brackish surface water and dense more saline bottom waters. Both water masses are separated by a stable halocline located at 50–80 water depth (Meier et al., 2006). The stratification leads to the establishment of suboxic, anoxic, or even euxinic conditions as dissolved oxygen is removed by the heterotrophic consumption of organic matter (Cederwall and Elmgren, 1990; Diaz and Rosenberg, 2008; Carstensen et al., 2014). As a consequence, the water column of the Baltic Sea contains strong salinity and oxygen gradients affecting the vertical zonation of microbial communities (Labrenz et al., 2010; Herlemann et al., 2011; Thureborn et al., 2013; Berg et al., 2015).

### 2.2. Sampling of the suspended particulate matter

Field work was conducted during expedition EMB201 on board R/V Elisabeth Mann Borgese in December 2018. Water temperature, conductivity, oxygen concentration and turbidity were measured using a SBE 911 CTD system (Seabird Electronics, United States). At three stations (Landsort Deep, Fårö Basin and EGB; Figure 1), SPM samples were collected at the surface, in the suboxic zone (O_2_ < 3 μM; Trouwborst et al., 2006), at the transition from the suboxic to the euxinic zone and in the proximity of the seafloor. Surface water (4 m water depth) was sampled using the clean water pumping system of the research vessel at all stations. Based on the oxygen concentration, sampling depths in the suboxic zone were determined individually at each site. The transition from the suboxic to the euxinic zone was chosen under consideration of increasing turbidity, presumably formed by the precipitation of colloidal sulfur particles (Schmale et al., 2016; Holtermann et al., 2017). In the EGB, water of the suboxic and euxinic zones were sampled with a pump-CTD (Strady et al., 2008). In the Fårö Basin and Landsort Deep, water was sampled with Niskin bottles and a giant water sampler (400 L; Duinker and Schulz-Bull, 1999), respectively. The water was stored in 1000 L tanks for subsequent filtration. The water was filtered with a flow rate of 1.3 to 1.5 L min^−1^ on pre-ashed, 142-mm-diameter, 0.7 μm pore size glass microfiber GF/F filters (Ahlstrom-Munksjö Germany), which were stored frozen at −20°C for later GDGT analysis. The amount of filtered water ranged between 150–600 L per sample. For DNA analysis, 1 L of water was filtered onto a 0.22 μm pore-size polycarbonate filter (Millipore GVWP, 47 mm diameter, Darmstadt, Germany), which was subsequently frozen in liquid nitrogen and stored at −80°C until analysis. For CARD-FISH analysis, 100 mL of water was fixed with particle-free formaldehyde (2% final concentration), incubated at 4°C for about 12 h before filtering onto 0.2 μm pore-size polycarbonate filters (Whatman 111106, 47 mm diameter) and stored at −80°C until analysis.

### 2.3. DNA-based analysis

DNA was extracted using the QIAmp DNA Mini Kit (Qiagen, Hilden, Germany). The filters were cut into small pieces and placed in a 1 mL microcentrifuge tube with 360 μL of buffer ATL. For all other steps the manufacturer’s procedures were followed. The DNA samples were sent to LGC Genomics GmbH (Berlin, Germany) for sequencing. There, for library preparation the primers A519F (S-D-Arch-0519-a-S-15) (5’-CAGCMGCCGCGGTAA-3′) and Bakt_805R (S-D-Bact-0785-a-A-21) (5’-GACTACHVGGGTATCTAATCC-3′), covering the V4 region, were used (Klindworth et al., 2013). Sequences were generated on an Illumina MiSeq in a 2×300 bp paired-endrun using V3 Chemistry (Illumina). After demultiplexing and primer clipping (performed by LGC Genomics), reads were further processed in dada2 version 1.20.0 (Callahan et al., 2016) implemented in R version 4.1.0 (R Core Team, 2021) to generate amplicon sequence variants (ASVs). Prior to denoising, forward and reverse reads were truncated to 140 and 200 bp, respectively, and filtered to a maximum expected error rate of 1. For error learning a modified error estimation function was used to avoid underestimation of error rates. Denoising, and merging of denoised paired-end reads were conducted with default settings, except that all reads were pooled for the denoising step. Chimera removal was performed with *removeBimeraDenovo()* using default settings. ASVs were taxonomically classified with *assignTaxonomy()* against the SILVA ribosomal reference database release 138.1 (Quast et al., 2013). ASVs present only once in the dataset, those shorter and longer than 250 bp and 252 bp, respectively, as well as those affiliated with eukaryotes and bacteria were removed from the data set. More details on amplicon sequence processing are available on.^1^

To explore the phylogenetic relationships among *Nitrosopumilus*, ASVs were placed in a phylogenetic tree reconstructed from full-length 16S rRNA gene sequences from available genome assemblies on NCBI refseq and genbank, and those extracted from metagenomic data sets available from the water column of the Baltic Sea (Supplementary Table S1). In total, 37 metagenomic data sets from three metagenomic sequencing projects (ENA project accessions: PRJEB22997, PRJEB34883, PRJNA273799) yielded full-length *Nitrosopumilus* 16S rRNA genes assembled with phyloFlash version 3.4 (Gruber-Vodicka et al., 2020). All sequences were then aligned against the SILVA ribosomal reference database release 138.1 (Quast et al., 2013) with SINA version 1.7.2 (Pruesse et al., 2012). After manual curation of the alignment (Supplementary Table S1), modeltest-ng version 0.1.7 (Darriba et al., 2020) and raxml-ng version 1.1.0 (Kozlov et al., 2019) were used to predict the best evolutionary model (TPM3 + I + G4) and infer a maximum likelihood tree with bootstrap support, respectively. Phylogenetic placement of *Nitrosopumilus* ASVs was conducted with epa-ng version 0.3.8 (Barbera et al., 2019), retaining only one placement per sequence. The final tree was visualized with iToL (Letunic and Bork, 2021).

### 2.4. Cell quantification

Catalyzed reporter deposition-fluorescence *in situ* hybridisation (CARD-FISH) was performed according to Pernthaler et al. (2002) with the following modifications. Filters were embedded in 0.1% agarose instead of 0.2% agarose. Before incubating the filters in the lysozyme solution, they were incubated in 0.01 M HCl for 10 min and washed in phosphate buffered saline (PBS) and Milli-Q water (MQ) afterwards. After lysozyme incubation (10 mg mL^−1^ in 0.05 M EDTA, 0.1 M Tris–HCl [pH 7.5]) for 60 min at 37°C, filters were washed in PBS and MQ. The second digestion was performed with 0.1 M HCl for 1 min. Filters were washed both in MQ and ethanol (EtOH) and air-dried before they were cut into pieces for hybridisation with the oligonucleotide probe Cren537 (5‘-TGACCACTTGAGGTGCTG-3′; targeting Crenarchaea; Teira et al., 2004). The hybridisation was performed at 35°C for 15 h in the presence of 35% formamide. The filters were washed in the preheated (37°C) washing buffer (420 μL 5 M NaCl, 500 μL 0.5 M EDTA [pH 7.4], 1,000 μL 1 M Tris–HCl [pH 7.4], filled up to 50 mL with MQ, 50 μL SDS) for 20–30 min at 37°C and incubated afterwards in PBST (500 μL 1 x PBS, 50 μL Triton X-100) for 45 min at 37°C. To remove excess liquid the filters were dabbed onto blotting paper before they were incubated in tyramide solution (1 mL amplification buffer, 10 μL 0.15% H_2_O_2_ [1,000 μL 1 x PBS, 5 μL 30% H_2_O_2_], 2 μL fluorescently labeled tyramide) for 30 min at 37°C in the dark. Again, the filters were dabbed onto blotting paper to remove excess water and incubated in PBST in the dark for 15 min. Then they were washed in MQ and EtOH in the dark and dried in the dark before they were embedded in Vectashield mounting medium (Vector Labs, Burlingame, CA, United States) containing 40,6-diamidin-2-phenylindol (DAPI). For enumeration by epifluorescence microscopy (Zeiss Axioskop 2 mot plus, Oberkochen, Germany) DAPI cells were counted on 0.001488 mm^2^ at 100-fold magnification, making up 300–700 DAPI-stained cells. Hybridized cells were counted from 15 randomly selected microscopic fields (0.01488 mm^2^).

### 2.5. IPL-GDGT analysis

The filters were lyophilized and one third of each filter was used for IPL-GDGT analysis. The filters were extracted using a modified Bligh and Dyer procedure (Bligh and Dyer, 1959). Briefly, the filters were extracted ultrasonically four times for 10 min in a solvent mixture of methanol (MeOH), dichloromethane (DCM) and phosphate buffer (2:1:0.8; v:v:v). After each sonification step, the supernatant was recovered by centrifugation at 1000 × g for 5 min. The combined supernatants were phase separated by adding DCM and phosphate buffer to a final solvent ratio of 1:1:0.9 (v:v:v). The organic phase, containing IPL-GDGTs, was collected and the aqueous phase re-extracted three times with DCM. The bulk of the solvent was removed under vacuum and the Bligh and Dyer extract dried under a gentle stream of N_2_. Before analysis, the extract was re-dissolved in a mixture of *n*-hexane:2-propanol:water (72:27:1; v:v:v) to a concentration of 10 mg mL^−1^ and aliquots were filtered through 0.45 μm regenerated cellulose syringe filters (4 mm diameter).

IPL-GDGTs were analysed by high-performance liquid chromatography coupled to tandem mass spectrometry (HPLC-MS^2^) following previously established protocols (Schouten et al., 2008). For this, an Alliance 2690 HPLC system (Waters, UK) coupled to a Quattro LC triple quadrupole mass spectrometer (Micromass, UK) was used. Separation of IPL-GDGTs was achieved using a Phenomenex Luna NH_2_ column (150 mm × 2 mm; 3 μm particle size) maintained at 30°C. The following linear elution gradient was used with a flow rate of 0.2 mL min^−1^: 100% eluent A to 35% eluent A–65% eluent B over 45 min, which was maintained for 20 min, and then back to 100% eluent A for 20 min to re-equilibrate the column, where eluent A = *n*-hexane/2-propanol/formic acid/14.8 M aqueous NH_3_ (79:20:0.12:0.04; v:v:v:v) and eluent B = 2-propanol/water/formic acid/14.8 M aqueous NH_3_ (88:10:0.12:0.04; v:v:v:v). The mass spectrometer was equipped with an electrospray ionization interface operated in positive ion mode. Source conditions were as described in Baumann et al. (2018). A mass range from *m/z* 600 to 2000 was monitored and IPL-GDGTs identified based on comparison with previously published mass spectral characteristics (Schouten et al., 2008; Sinninghe Damsté et al., 2012). The peak areas were determined from extracted ion chromatograms of the [M + H]^+^, [M + NH4]^+^ and [M + Na]^+^ for each individual IPL-GDGT species. As no standards were available, IPL-GDGTs were examined in terms of their peak area response. It should be noted that the relative abundance of the peak area does not necessarily reflect the actual abundance of the different IPL-GDGTs due to different ionization efficiencies.

### 2.6. Core GDGT analysis

Another third of the filters were extracted by ultrasonication three times with MeOH, DCM/MeOH (1:1; v:v) and DCM. After each extraction step, the samples were centrifuged, and the extracts combined into a total lipid extract (TLE). Each TLE was spiked with 100 μL C_46_ GDGT (0.01 μg/μl) as internal standard. The TLEs were separated into four fractions by column chromatography with silica gel as solid phase and *n*-hexane, *n*-hexane/DCM (1:1; v:v), DCM and DCM/MeOH (1:1; v:v) as respective eluents. The DCM/MeOH fractions, containing the core GDGTs, were filtered with a 0.45 μm PTFE filter before being analysed by HPLC coupled to atmospheric pressure chemical ionization mass spectrometry (APCI/MS) using a ThermoScientific Dionex Ultimate 3000 UHPLC system coupled to a ThermoScientific MSQ Plus as described in Kaiser and Arz (2016) except for the following modification. After Hopmans et al. (2016), separation of individual core GDGTs was achieved on two UHPLC silica columns (BEH HILIC, 2.1 mm x 150 mm, 1.7 μm; Waters) in series, fitted with a pre-column of the same material, which were all maintained at 30°C. Using a flow rate of 0.2 mL min^−1^, the gradient of the mobile phase was first held isocratic for 25 min with 82% solvent A (*n*-hexane) and 18% solvent B (*n*-hexane:2-propanol, 9:1, v:v), followed by a linear gradient to 35% solvent B in 25 min and a linear gradient to 100% solvent B in 30 min. Thereafter, the column was re-equilibrated with 18% solvent B for 20 min. Isoprenoid GDGTs were identified in selected ion monitoring (SIM) mode as described in Hopmans et al. (2004) and Schouten et al. (2007a,b).

## 3. Results

### 3.1. Physicochemical conditions

**Figure 2:**
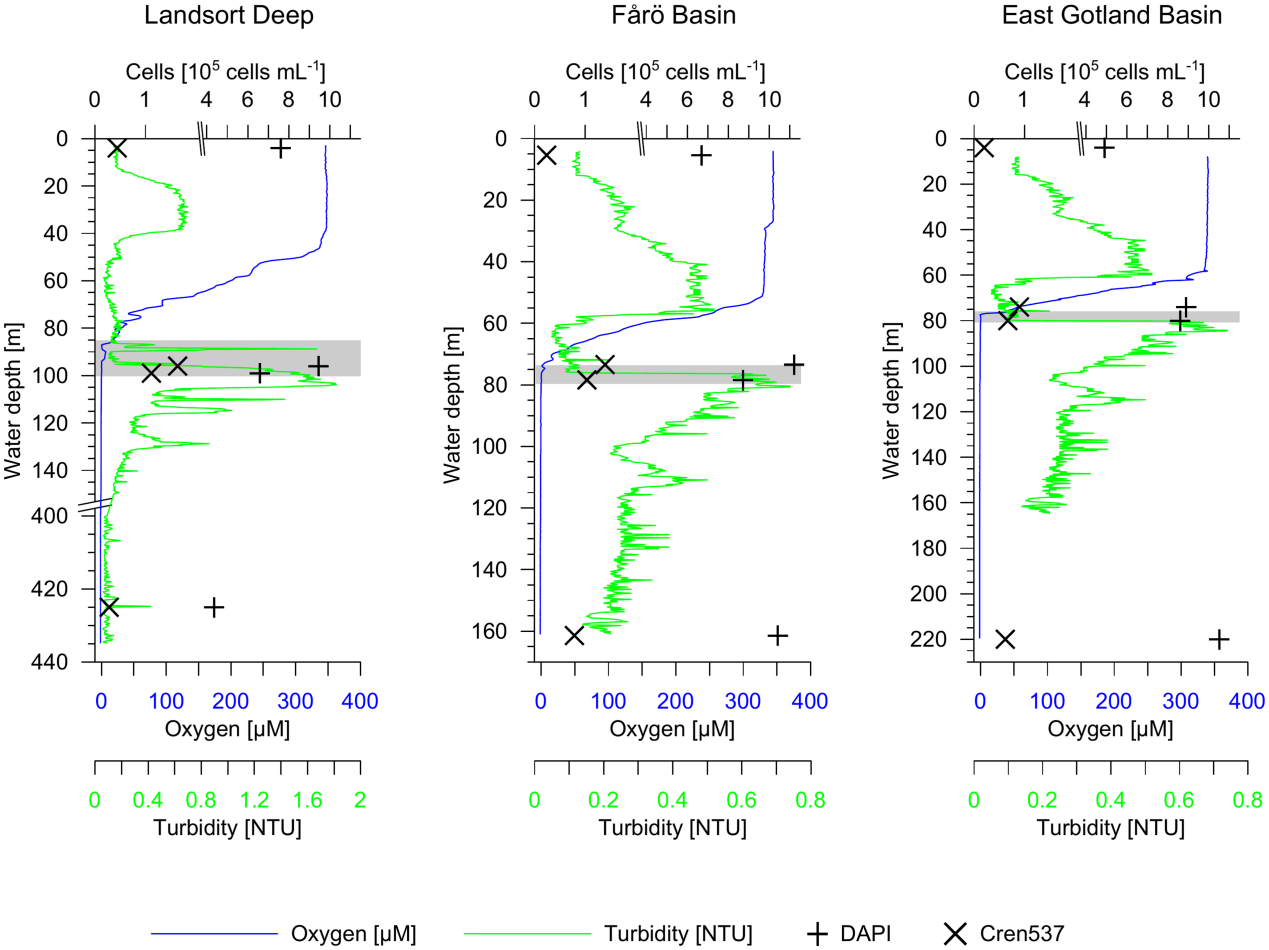
Oxygen concentration, turbidity and microbial cells in water column profiles of the Landsort Deep, Fårö Basin and East Gotland Basin. Total 40,6-diamidin-2-phenylindol (DAPI) and Nitrosospheria cell concentrations are shown. The grey horizontal bars highlight the suboxic zone at each site.

In the Landsort Deep, the water was suboxic below 86 m (Figure 2). The oxygen concentration decreased irregularly probably due to weak lateral inflows increasing the oxygen content in the suboxic zone. The turbidity increased at 100 m and marked the transition to the euxinic zone. In the EGB, the suboxic zone was located between 74–79 m and between 77–80 m in the Fårö Basin (Figure 2).

### 3.2. Total microbial (DAPI) and crenarchaeota (Cren537) cell counts

In the Landsort Deep, the total microbial cell concentration was 7.60 × 10^5^ cells mL^−1^ at the surface, 9.44 × 10^5^ cells mL^−1^ in the suboxic zone and only 4.35 × 10^5^ cells mL^−1^ in the euxinic zone (Figure 2). Total microbial cell abundances increased from the surface (6.68 and 4.90 × 10^5^ cells mL^−1^) toward the euxinic zone (1.04 and 1.05 × 10^6^ cells mL^−1^) in both the Fårö Basin and the EGB (Figure 2). The concentration of crenarchaeotal cells represented a low fraction of the total prokaryotic community (3–4% at the surface; 7–15% at the suboxic zone and 6–7% at the euxinic zone according to CARD-FISH data). The concentration of crenarchaeotal cells was lowest in surface waters (0.2–0.4 × 10^5^ cells mL^−1^; Figure 2), except for the Landsort Deep with lowest concentrations in the euxinic zone. It was highest in the suboxic zone with values around 1.0–1.6 × 10^5^ cells mL^−1^ in the Landsort Deep and the Fårö Basin, and 0.7–0.9 × 10^5^ cells mL^−1^ in the EGB. In the euxinic zone, the cell concentrations decreased again to 0.3–0.8 × 10^5^ cells mL^−1^ in the Landsort Deep and the Fårö Basin. The decrease in cell concentrations was less pronounced in the euxinic zone (0.6 × 10^5^ cells mL^−1^) compared to the suboxic zone of the EGB.

### 3.3. Diversity of archaeal groups

In all basins and water depths, the class Nitrososphaeria, represented solely by the genus *Nitrosopumilus*, dominated the archaeal community at the surface (98–99% of all archaeal sequences), in the suboxic zone (83–100%), and in the euxinic zone (88–93%; Table 1). Woesarchaeales occurred mainly in the suboxic and euxinic zones of the Landsort Deep (10–17%) and the Fårö Basin (4–7%), and in the euxinic zone of the EGB (9%). Marine Group II archaea were found at the surface of the Landsort Deep and the Fårö Basin (1–2%) and in the suboxic zones of all basins (<1%). Methanofastidiosales (class Thermococci) were present in the euxinic zones of the Fårö Basin and the EGB (<1%). Some reads of Bathyarchaea occured in the EGB euxinic zone (<1%). Thus, the CARD-FISH probe Cren537 presumably detected mainly Nitrosopumilus in the samples.

**Table 1:** Archaeal community composition expressed as percentages of the total archaeal sequencing reads at different water depths of the Landsort Deep, the Fårö Basin and the East Gotland Basin.

Phylum	Class	Order	Landsort Deep (water depth in m)				Fårö Basin (water depth in m)				East Gotland Basin (water depth in m)			
			4	96	99	425	4	72	77	160	4	74	80	220
Altiarchaeota	Altiarchaeia	Altiarchaeales	0.00	0.00	0.00	0.00	0.00	0.00	0.00	0.00	0.00	0.00	0.00	0.07
Asgardarchaeota	Asgardarchaeota		0.00	0.00	0.00	0.03	0.00	0.00	0.00	0.03	0.00	0.00	0.00	0.01
Crenarchaeota	Bathyarchaeia		0.00	0.04	0.00	0.03	0.00	0.00	0.00	0.03	0.00	0.00	0.00	0.26
Iainarchaeota	Iainarchaeia	Iainarchaeales	0.00	0.34	0.22	0.27	0.00	0.00	0.00	0.45	0.00	0.00	0.00	1.30
Nanoarchaeota	Nanoarchaeia	Woesearchaeales	0.00	16.77	14.55	10.32	0.09	3.51	4.15	6.53	0.16	0.06	0.70	9.36
Crenarchaeota	Nitrososphaeria	Nitrosopumilales	98.93	82.73	85.12	89.28	98.12	96.42	95.80	92.64	99.19	99.71	99.29	88.36
Euryarchaeota	Thermococci	Methanofastidiosales	0.00	0.04	0.06	0.02	0.09	0.00	0.00	0.30	0.00	0.00	0.02	0.61
Thermoplasmatota	Thermoplasmata	Marine Group II	1.07	0.08	0.00	0.00	1.70	0.06	0.05	0.00	0.65	0.23	0.00	0.00
Thermoplasmatota	Thermoplasmata		0.00	0.00	0.00	0.00	0.00	0.00	0.00	0.02	0.00	0.00	0.00	0.03
Thermoplasmatota			0.00	0.00	0.06	0.05	0.00	0.00	0.00	0.00	0.00	0.00	0.00	0.00

Unclassified taxa were left blank at the respective rank.

### 3.4. Phylogenetic placement of *Ca.* Nitrosopumilus

Reconstructing the phylogenetic relationships within the genus *Nitrosopumilus* based on full length 16S sequences proved inconclusive, as most branches displayed a very low bootstrap support. This is probably because the 16S rRNA gene is not well-suited to resolve the phylogeny below genus level for *Nitrosopumilus,* as has been previously documented (Alves et al., 2018; Wang et al., 2021).

**Figure 3:**
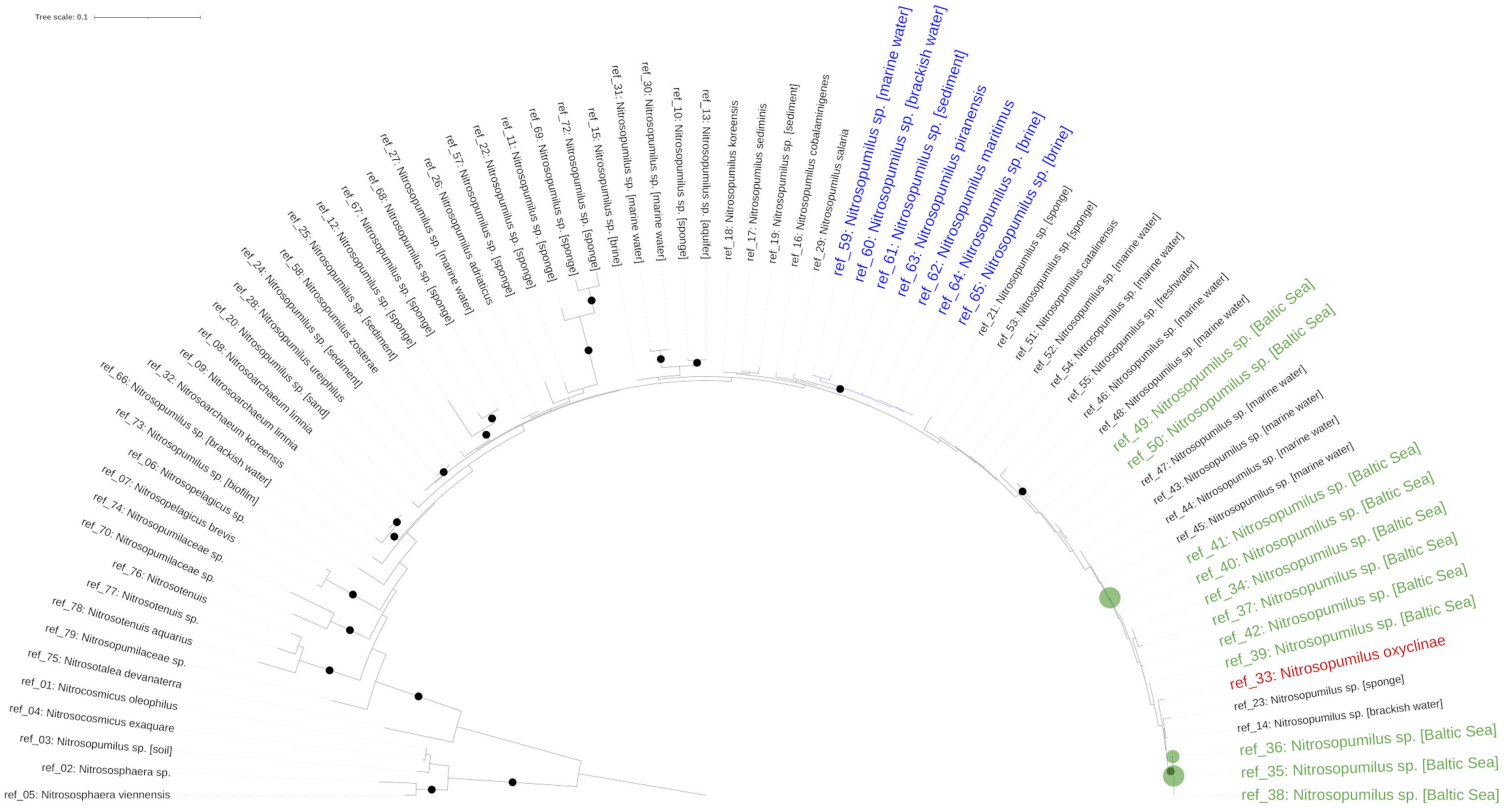
16S rRNA-based phylogenetic tree. The green dots mark the position of the *Nitrosopumilus* ASVs. The position of *N. oxyclinae* is highlighted in red. The well supported cluster of *N. maritimus* is marked in blue.

In turn, this made the phylogenetic placement of the detected ASVs below genus level difficult. Only the cultured representatives of *N. maritimus* and *N. piranensis* as well as *Nitrosoarchaeum* were located on well supported branches (Figure 3). Our detected ASVs were placed in an unresolved part of the phylogenetic tree containing metagenome-reconstructed 16S data from the Baltic Sea and the cultured representative of *N. oxyclinae*. The ASVs were either identical or had at most one mismatch to reconstructed 16S sequences originating from the Baltic Sea, whereas similarity to all described cultured representatives, including *N. maritimus*, was lower (≥2 mismatches; Supplementary Table S2).

### 3.5. Archaeal lipid abundance and distribution

**Figure 4:**
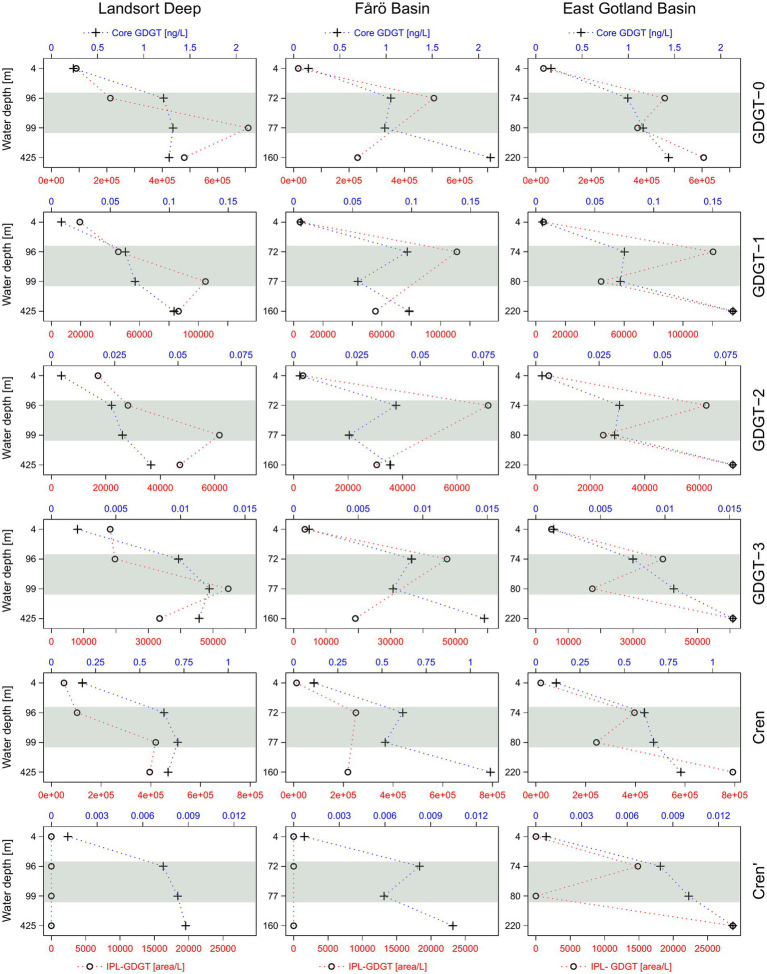
Relative abundance of intact (IPL) and concentration of core GDGT-1, GDGT-2, GDGT-3, crenarchaeol and its isomer (cren’) in the water column of the Landsort Deep, the Fårö Basin and the East Gotland Basin. The grey horizontal bars highlight the suboxic zone at each site.

**Figure 5:**
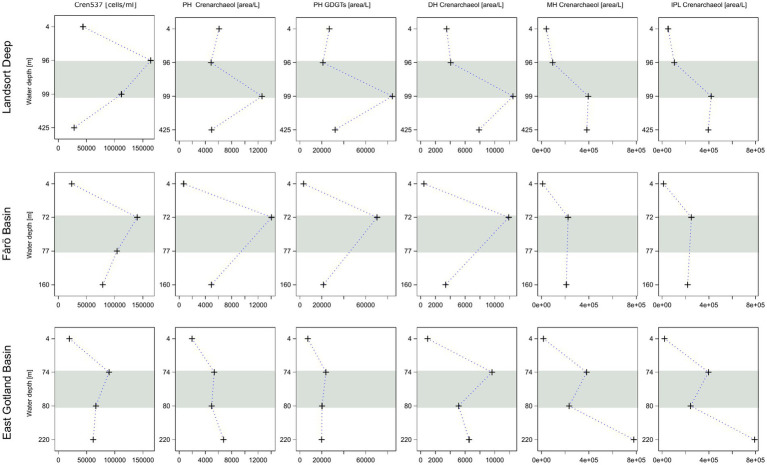
Concentrations of Nitrososphaeria cells compared to the relative abundance of phosphohexose (PH) crenarchaeol, the sum of PH GDGTs, dihexose (DH) and monohoexose (MH) crenarchaeol, and the sum of PH, DH and MH crenarchaeol in the water column of the Landsort Deep, the Fårö Basin and the East Gotland Basin. The grey horizontal bars highlight the suboxic zone at each site.

**Figure 6:**
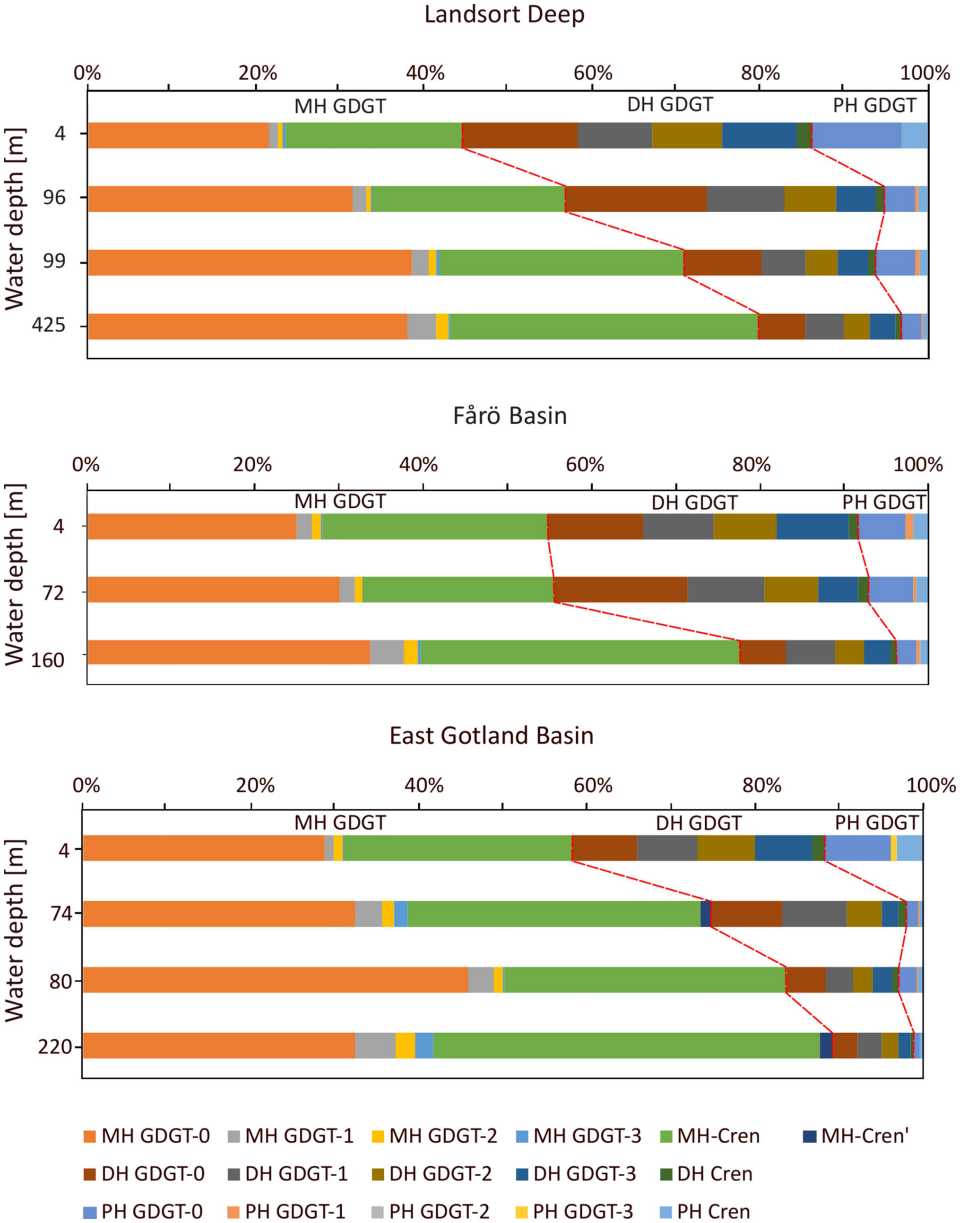
Relative abundance of monohoexose (MH), dihexose (DH) and phosphohexose (PH) GDGTs in the water column of the Landsort Deep, the Fårö Basin and the East Gotland Basin.

Core GDGTs included GDGT-0 to −3, crenarchaeol and its isomer (cren’). The same suite of GDGTs was detected as IPLs with monohexose (MH), dihexose (DH) and phosphohexose (PH) headgroups. Core GDGTs showed increasing concentrations from the surface to the euxinic zones in all three basins (Figure 4). The abundance of IPL-GDGTs increased from the surface water to the suboxic zone and decreased again in the euxinic zones of the Landsort Deep and the Fårö Basin, whereas they increased in the euxinic zone of the EGB. The highest abundances of PH-crenarchaeol and the cumulative abundance of PH-GDGTs were found in the suboxic zone (Figure 5), except for the EGB. The relative abundance of DH-GDGTs and PH-GDGTs decreased with increasing depth (from 39 to 18% and 11 to 3%, respectively), while the relative abundance of MH-GDGTs increased with depth (from 50 to 79%; Figure 6) in the Landsort Deep and the Fårö Basin. The relative abundances of DH-GDGTs and PH-GDGTs were lower in the EGB in comparison to the other two basins and decreased also with depth (from 30 to 9% and 12 to 1%, respectively). Thus, the percentage of MH-GDGTs was higher in the EGB (58 to 89%), compared to the two other basins.

## 4. Discussion

### 4.1. Potential GDGT source organisms in the Central Baltic Sea

The archaeal community composition of the central Baltic Sea appears unique due to the dominance of *Nitrosopumilus* throughout the water column. Although our data represent winter conditions in the central Baltic Sea, the results are supported on a larger temporal and spatial scale by data mining of metagenomes (Hugerth et al., 2015; Alneberg et al., 2018, 2020), showing *Nitrosopumilus* as most prevalent representative of Nitrososphaeria for different seasons and locations of the Baltic Sea (Supplementary Figure S3). In contrast, the archaeal distribution in the Black Sea water column, which is also characterized by a pronounced oxycline and euxinic bottom waters, is more variable (Sollai et al., 2019). The oxic zone is dominated by *Ca.* Nitrosopelagicus, which we did not detect in the Baltic Sea. The suboxic zone is dominated by *N. maritimus*, which is a species within the genus *Nitrosopumilus*. Thus, the dominating genus is the same as in the Baltic Sea suboxic zone. The euxinic zone is dominated by Thermoplasmatales, Bathyarchaeota and Woesarchaeales. All three orders were also found in the Baltic Sea, but in lower proportions (Table 1).

Nitrososphaeria, represented exclusively by *Nitrosopumilus*, is the dominant archaeal class (82 to 100%) and it is therefore suggested to be the main source of GDGTs in the central Baltic Sea. The high abundance of IPL and core crenarchaeol (together with its isomer; Figure 4), which is known to be produced exclusively by Crenarchaeota (Schouten et al., 2008; Sinninghe Damsté et al., 2012), confirms its dominance by the lipidomic approach. Other potential GDGT producers, such as Methanofastidiosales or Thermoplasmata (phylum: Thermoplasmatota, belonging formerly to the phylum Euryarchaeota; Zeng et al., 2022), occurred only in very low proportions (<2%) throughout the water column (Table 1). Therefore, they most likely do not contribute significantly to the GDGT pool. Previous studies in the central Baltic Sea (Labrenz et al., 2010; Thureborn et al., 2013) found that Nitrososphaeria was most abundant at the redoxcline in the deeper water column. However, due to the insufficient taxonomic resolution, it is unclear whether these studies detected the same genus as in the present study.

Woesearchaeales, which were found in the suboxic and euxinic zones of all three basins, lack essential enzymes related to GDGT membrane lipid biosynthesis (Villanueva et al., 2017), although one protein required for the production of GDGTs has been identified recently (Zeng et al., 2022). These microorganisms possess the ability to recycle GDGTs from dead cells to compensate for their metabolic deficiencies and build their cellular membranes (Lipsewers et al., 2018; Liu et al., 2018). Such recycled GDGTs cannot be detected in the core GDGT pool of the water column as they are moved into the IPL-GDGT pool conserving an IPL-GDGT signal produced by other organisms potentially thriving at different depths. Thus, it appears unlikely that Woesarchaeales contribute significantly to GDGT synthesis in the Baltic Sea.

### 4.2. Factors influencing the distribution of IPL and core GDGTs in the central Baltic Sea

Core GDGTs concentrations are generally increasing with depth in all three basins (Figure 4). This is to be expected as core GDGTs represent degradation products of IPL-GDGTs and accumulate toward the bottom. In contrast, most IPL-GDGTs show highest concentrations in the suboxic zones of the Landsort Deep and the Fårö Basin with the exception of MH-crenarchaeol (Figure 5). The latter might be derived originally from hexose-phosphohexose (HPH) crenarchaeol (Schouten et al., 2012). As MH is the dominant headgroup of IPL-GDGTs, its depth trend determines the general trend of the summed IPL-crenarchaeol (Figure 5).

The dominance of *Nitrosopumilus* as principle GDGT source allows evaluating how the distribution of IPL (and core) GDGTs is influenced by parameters other than community composition. One of these parameters is the variable degradation rate of IPL-GDGTs. Ester phospholipids degrade under aerobic conditions 20 times faster than glycosidic ether lipids (Harvey et al., 1986; Schouten et al., 2010). Thus, phospholipids are deemed to be most appropriate markers for living archaea, beside cell counting. For example, HPH-GDGTs and to a lesser degree DH-crenarchaeol, common phospholipids in environmental samples (Besseling et al., 2019; Sollai et al., 2019), have been postulated to trace the abundance of active ammonia-oxidizing archaea (Pitcher et al., 2011a; Schouten et al., 2012).

Surprisingly, HPH-GDGTs were not found in the water column of the central Baltic Sea. Instead, PH-GDGTs were found with maximum abundances in the suboxic zone, in agreement with observations based on cell abundances (Figure 5). PH-GDGTs were reported until now only in culture experiments (Schouten et al., 2008; Elling et al., 2017). Those studies showed that the suite of IPL-GDGTs varied among crenarchaeal strains. For example, PH-GDGT 0–4 occurred exclusively in Group 1.1a, the *Nitrosopumilus* lineage, while HPH-GDGTs were abundant only in some strains of marine Group 1.1a and common in the Nitrososphaera lineage (Elling et al., 2017). Furthermore, the fact that DH-crenarchaeol shows a profile similar to that of PH-GDGTs suggests that the Baltic Sea *Nitrosopumilus* may indeed produce PH-GDGTs instead of HPH-GDGTs. Thus, the enhanced PH-GDGT abundance in the suboxic zone marks the ecological niche of *Nitrosopumilus* in the central Baltic Sea in agreement with previous microbiological studies (Labrenz et al., 2010; Berg et al., 2015).

The phylogenetic evidence provided in this study indicates that the Baltic Sea *Nitrosopumilus* is different from the well supported clade of the cultured representatives (*N. maritimus*, *N. piranensis*), but most closely related to *N. oxyclinae* (Figure 3). Despite lacking support, our phylogeny agrees with a recently published genome-based phylogenetic tree of the class Nitrososphaeria (Qin et al., 2020; Ren and Wang, 2022) and a phylogenetic tree based on the ammonia monooxygenase (amoA) gene, a maker gene for archaeal nitrification (Alves et al., 2018). In these trees, *N. maritimus* and *N. piranensis* are located on a well-supported sister clade of *N. oxyclinae*, confirming our 16S rRNA gene-based phylogeny and thus supporting our hypothesis that Baltic Sea *Nitrosopumilus* may share a common ancestor with *N. oxyclinae*. This latter, which has the ability to grow in a wide salinity (10–40‰) and temperature (4–30°C) range, was isolated from the 175 m deep Hood Canal, Canada (Qin et al., 2017). This fjord has a similar setting than the central Baltic Sea in terms of stratification and poor ventilation as a sill limits the exchange with saline and oxygen rich water from the Admiralty Inlet (Paulson et al., 2006; Gregg and Pratt, 2010).

The IPL-GDGT profiles from the EGB are different from those in the other basins. The suboxic zone contains a lower concentration of PH crenarchaeol and a lower sum of PH-GDGTs in combination with lowest crenarchaeal cell counts (Figure 5). These data imply a reduced occurrence of GDGT producing Nitrososphaeria in the suboxic zone of the EGB compared to the Landsort Deep and Fårö Basin. In contrast, the summed IPL-GDGTs are abundant at the euxinic zone with highest proportions of MH-GDGTs and lowest proportions of DH-GDGTs (Figure 6). MH crenarchaeol, the most abundant IPL-GDGT, may be a degradation product of PH crenarchaeol and might have accumulated near the bottom of the EGB due to lateral inflows (Dargahi and Cvetkovic, 2014). Additionally, Woesarchaeales might increase the IPL-GDGT pool, as they conserve GDGTs produced by other organisms against decay (Lipsewers et al., 2018).

### 4.3. Significance for GDGT-based indices

As GDGTs in the water column of the Baltic Sea are produced almost exclusively by *Nitrosopumilus*, the variability of different GDGT-based indices within a single species can be estimated (Table 3).

First, the GDGT-2/GDGT-3 ratio, an indicator for shallow versus deep-dwelling archaeal community (Taylor et al., 2013; Kim et al., 2015), is relatively stable around 1.3 ± 0.24 (mean and standard deviation) for IPL-GDGTs. The values range between 1.8 and 5.1 and tend to increase with depth for core GDGTs. Preferential bondage and degradation most likely account for the increasing trend of the core GDGT-based ratio. A potential faster degradation of DH-GDGT-2 compared to MH-GDGT-2 (Figure 6) may explain lower values of the GDGT-2/GDGT-3 for core GDGTs. Similarly, a decrease of GDGT-2/GDGT-3 ratio in the MH-GDGT fraction from the suboxic zone to the euxinic zone suggests a faster degradation of GDGT-2 compared to GDGT-3, and thus lower GDGT-2/GDGT-3 ratios of core GDGTs. Second, the GDGT-0/cren ratio, an indicator for the presence of methanogenic archaea (Blaga et al., 2009), is below 2 within both IPL (1.46 ± 0.38) and core (1.69 ± 0.2) GDGTs (Table 3). This value suggests also that methanogenic archaea do not occur in the water column, in agreement with the 16S rRNA sequencing results. Third, the methane index (MI), an indicator for the presence of methanotrophic Euryarchaea (Zhang et al., 2011), is below 0.3 for both IPL and core GDGTs (0.21 ± 0.05 and 0.14 ± 0.05, respectively) and implies an absence of archaeal methanotrophs in the investigated basins, corroborating the 16S rRNA sequencing results.

The ${\mathrm{TEX}}_{86}^{L}$ temperature proxy (Eqs 1), which represents a TEX_86_ derivative adapted to comparatively low water temperatures (<15°C; Kim et al., 2010), has been calibrated for the Baltic Sea, although it is currently under debate whether it reflects summer surface (Kabel et al., 2012) or annual subsurface temperatures (Wittenborn et al., 2022).

(1) $${\mathrm{TEX}}_{86}^{L}=\log\left(\frac{GDGT-1}{\left(GDGT-1+GDGT-2+GDGT-3\right)}\right)$$

However, in agreement with other investigations (Labrenz et al., 2010; Thureborn et al., 2013; Berg et al., 2015), the present study suggests that GDGT-producers are most abundant at depth within the suboxic zone of the Baltic Sea. Therefore, we applied the subsurface calibration established by Wittenborn et al. (2022) to convert ${\mathrm{TEX}}_{86}^{L}$ values based on either core GDGTs, summed IPL-GDGTs, MH-GDGTs and DH-GDGTs into water temperatures (Table 2). As IPL-GDGTs reflect living cells, a higher accordance is expected between IPL-GDGTs-derived temperature estimates and *in situ* temperatures compared to core GDGT-based temperatures. Indeed, core GDGT-derived temperatures are *ca.* 1–2°C lower than measured temperatures. This offset can be explained by a preferred bondage between core GDGTs and specific headgroups. GDGT-0, GDGT-1 and crenarchaeol are preferentially bonded to PH and HPH headgroups, while GDGT-2 and GDGT-3 are preferentially coupled to MH and DH headgroups (Pitcher et al., 2011b). Considering a faster degradation rate for PH-GDGTs than for MH-and DH-GDGTs (Harvey et al., 1986; Schouten et al., 2010), GDGT-1 may be more abundant in the core GDGT fraction and the resulting ${\mathrm{TEX}}_{86}^{L}$ is biased toward lower temperature estimates. Summed IPL-GDGT-based ${\mathrm{TEX}}_{86}^{L}$ temperatures are in close agreement (average of the absolute deviation ±0.7°C) with measured temperatures, although the water temperature in euxinic waters is always underestimated by *ca.* 1–2°C. Considering that members of the Nitrososphaeria need oxygen for their metabolism (Offre et al., 2013), the archaeal cells in the euxinic zone may reside inactive (Berg et al., 2015). The IPL-GDGT signal may thus represent the temperature of the original habitat depth, i.e., the lower water temperature of the suboxic zone. It is striking that ${\mathrm{TEX}}_{86}^{L}$ temperature estimates based on MH-GDGTs deviates strongly (average of the absolute deviation ±1.7°C) from measured temperatures, while DH-GDGTs derived temperatures are closer (average of the absolute deviation ±0.8°C) to *in situ* temperatures. This may again be related to the variable abundance of core GDGTs with different headgroups. The MH headgroup is tied about twice as often to GDGT-1 than to GDGT-2 and very rarely to GDGT-3, whereas the DH headgroup is almost evenly bound to GDGT-1, GDGT-2, and GDGT-3 (Figure 6). Thus, GDGT-1 is overrepresented in the MH-GDGT-based ${\mathrm{TEX}}_{86}^{L}$ reconstructions, resulting in temperatures biased toward lower estimates. In addition, the MH-GDGT fraction might also contain degradation products of the PH-GDGT fraction as mentioned above. *In situ* temperature is therefore most accurately reflected by ${\mathrm{TEX}}_{86}^{L}$ using the summed abundance of IPL-GDGTs. This is surprising as the sedimentary downcore calibration from Wittenborn et al. (2022) may not be directly suitable for SPM data. Still the IPL-derived ${\mathrm{TEX}}_{86}^{L}$ temperatures are very similar to observed temperatures.

**Table 2:** Measured and $\;{\mathrm{TEX}}_{86}^{L}$ -derived water temperatures in the water column of the Landsort Deep, the Fårö Basin and the Eastern Gotland Basin (EGB).

	Water depth (m)	Water temperatures (°C)				
		observed	${\mathrm{TEX}}_{86}^{L}$ IPL	${\mathrm{TEX}}_{86}^{L}$ MH	${\mathrm{TEX}}_{86}^{L}$ DH	${\mathrm{TEX}}_{86}^{L}$ Core
Landsort Deep	4	6.1	6.6	4.3	6.7	3.9
	96	5.5	6.1	5.6	6.3	3.9
	99	5.5	5.3	3.7	6.0	4.1
	425	6.2	5.4	5.3	5.7	4.2
Fårö Basin	4	6.2	6.3	9.2	6.3	4.2
	72	5.3	6.5	6.1	6.8	5.2
	160	6.7	5.7	5.7	5.6	4.1
EGB	4	6.3	6.9	9.6	6.9	4.3
	74	4.8	5.4	3.4	6.2	5.5
	80	5.4	5.5	3.8	6.6	5.1
	220	6.9	4.9	3.8	6.5	6.1

${\mathrm{TEX}}_{86}^{L}$ temperature estimates were based on the sum of IPL-GDGTs, monohoexose (MH) GDGTs, dihexose (DH) GDGTs and core GDGTs. ${\mathrm{TEX}}_{86}^{L}$ is defined as: ${\mathrm{TEX}}_{86}^{L}$ = log((GDGT-2) / (GDGT-1 + GDGT-2 + GDGT-3)) (Kim et al., 2010). The calibration according to Wittenborn et al. (2022) was applied to convert ${\mathrm{TEX}}_{86}^{L}$ values into subsurface water temperature (T = 26.21 * ${\mathrm{TEX}}_{86}^{L}$ + 19.79).

**Table 3:** Core and IPL-GDGT based methane index (MI), GDGT-0/cren, GDGT-2/GDGT-3 values in the water column of the Landsort Deep, the Fårö Basin and the Eastern Gotland Basin (EGB).

	Water depth [m]	MI-Index		GDGT-0/GDGT-cren		GDGT-2/GDGT-3	
		IPL	Core	IPL	Core	IPL	Core
Landsort Deep	4	0.3	0.1	1.8	1.4	0.9	1.9
	96	0.2	0.1	2.1	1.9	1.4	2.4
	99	0.2	0.1	1.7	1.8	1.1	2.3
	425	0.2	0.2	1.2	1.9	1.4	3.4
Fårö Basin	4	0.3	0.1	1.4	1.4	1.0	2.1
	72	0.2	0.2	2.0	1.7	1.5	4.4
	160	0.2	0.1	1.1	1.9	1.6	2.6
EGB	4	0.2	0.1	1.4	1.4	1.0	1.8
	74	0.2	0.2	1.2	1.6	1.6	4.4
	80	0.1	0.1	1.5	1.7	1.4	2.9
	220	0.2	0.2	0.8	1.8	1.2	5.1

The methane index is defined as: MI = (GDGT-1 + GDGT-2 + GDGT-3) / (GDGT-1 + GDGT-2 + GDGT-3 + crenarchaeol + cren’) (Zhang et al., 2011).

## 5. Conclusion

*Nitrosopumilus* is ubiquitously distributed in the water column of the central Baltic Sea and the most dominant biological source of GDGTs. Highest abundances of IPL-GDGTs and cell counts within the suboxic zone provide evidence that *Nitrosopumilus* resides primarily in the deeper water column and that the ${\mathrm{TEX}}_{86}^{L}$ reflect mainly subsurface water temperatures. Interestingly, members of *Nitrosopumilus* from the central Baltic Sea produce PH-GDGTs instead of HPH-GDGTs, which suggests that the Baltic Sea *Nitrosopumilus* may either differ from other species, or that the offset in the lipid distribution pattern is a physiological adaptation to the brackish conditions of the Baltic Sea. 16S rRNA analysis points to *N. oxyclinae* as the closest relative to the Baltic Sea *Nitrosopumilus*. A full metagenomic analysis is required to determine the exact phylogenetic position of the Baltic Sea *Nitrosopumilus*.

## Data availability statement

Sequence data for this study have been deposited in the European Nucleotide Archive (ENA) at EMBL-EBI under accession number PRJEB61728 (https://www.ebi.ac.uk/ena/data/view/PRJEB61728), using the data brokerage service of the German Federation for Biological Data (GFBio, Diepenbroek et al., 2014), in compliance with the Minimal Information about any (X) Sequence (MIxS) standard (Yilmaz et al., 2011). Lipid and CARD-FISH data were achieved at PANGAEA (Wittenborn et al., 2023).

## Author contributions

AW, JK, HA, and KJ contributed to conception and design of the study. AW, TB, CH, KK, and JW-R contributed to laboratory and analytical work. AW and CH organized the database. AW wrote the first draft of the manuscript. TB and CH wrote sections of the manuscript. All authors contributed to the article and approved the submitted version.

## Funding

This work was supported by the German Research Foundation (DFG) [grant KA 3228/2–1] and the Leibniz Association [grant number SAW-2017-IOW-2].

## Conflict of interest

The authors declare that the research was conducted in the absence of any commercial or financial relationships that could be construed as a potential conflict of interest.

## Publisher’s note

All claims expressed in this article are solely those of the authors and do not necessarily represent those of their affiliated organizations, or those of the publisher, the editors and the reviewers. Any product that may be evaluated in this article, or claim that may be made by its manufacturer, is not guaranteed or endorsed by the publisher.

## Footnotes

^1^ https://git.io-warnemuende.de/bio_inf/IOWseq000013

## Supplementary material

SUPPLEMENTARY TABLE S1 Multiple sequence alignment used for the reconstruction of the phylogenetic tree. Accession numbers and information about redundant sequences is provided.

SUPPLEMENTARY TABLE S2 Sequence similarity between *Nitrosopumilus* ASVs and full length 16S rRNA sequences used for phylogenetic tree reconstruction.

SUPPLEMENTARY TABLE S3 Map of 211 metagenomes of environmental DNA from the Baltic Sea. Black dots represent positions from which full length *Nitrosopumilus* 16S rRNA could not be extracted, whereas green dots represent positions where full length *Nitrosopumilus* 16S rRNA could be extracted (in total 37 metagenomes). Titles include depth and date of sampling. No other Nitrososphaeria representatives were found with the exception of one partial Nitrosopelagicus 16S rRNA sequence, reconstructed from a metaG of the Skagerrak.
