# Supplementary figures and images for: Nitrosopumilus as main source of isoprenoid glycerol dialkyl glycerol tetraether lipids in the central Baltic Sea
Source: Front Microbiol. 2023 Sep 28;14:1216130. doi: 10.3389/fmicb.2023.1216130 (PMC10575479; doi:10.3389/fmicb.2023.1216130)

Latitude

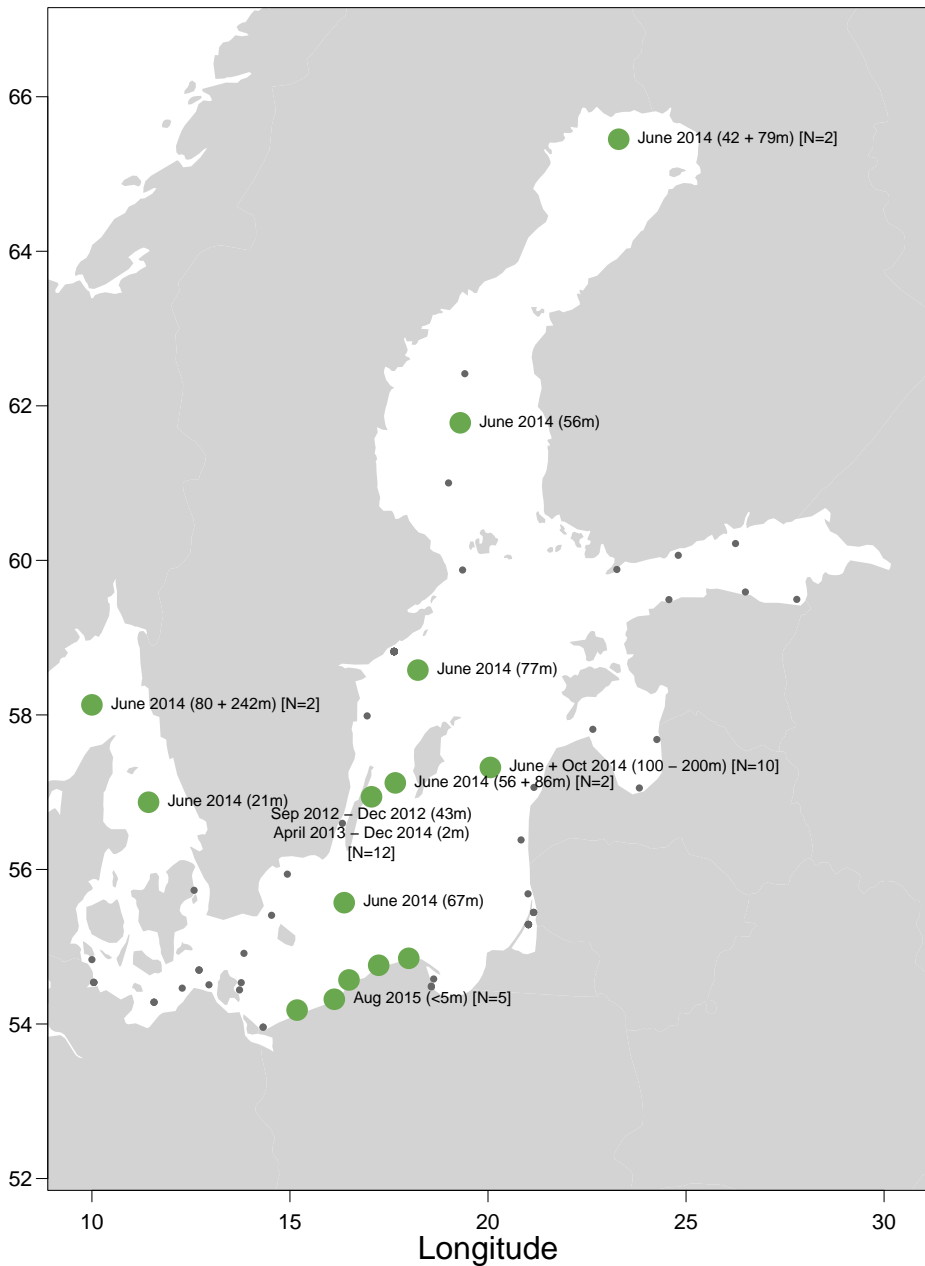

Supplement: SUPPLEMENTARY TABLE S3 — Map of 211 metagenomes of environmental DNA from the Baltic Sea. Black dots represent positions from which full length Nitrosopumilus 16S rRNA could not be extracted, whereas green dots represent positions where full length Nitrosopumilus 16S rRNA could be extracted (in total 37 metagenomes). Titles include depth and date of sampling. No other Nitrososphaeria representatives were found with the exception of one partial Nitrosopelagicus 16S rRNA sequence, reconstructed from a metaG of the Skagerrak. [file Data_Sheet_1.PDF]
